# How to have more things by forgetting how to count them^†^

**DOI:** 10.1098/rspa.2019.0782

**Published:** 2020-07-29

**Authors:** Asaf Karagila, Philipp Schlicht

**Affiliations:** 1 School of Mathematics, University of East Anglia, Norwich NR4 7TJ, UK; 2 School of Mathematics, University of Bristol, Fry Building, Woodland Road, Bristol BS8 1UG, UK

**Keywords:** symmetric extensions, Cohen’s first model, Axiom of Choice, Cohen forcing, Dedekind-finite sets

## Abstract

Cohen’s first model is a model of Zermelo–Fraenkel set theory in which there is a Dedekind-finite set of real numbers, and it is perhaps the most famous model where the Axiom of Choice fails. We force over this model to add a function from this Dedekind-finite set to some infinite ordinal *κ*. In the case that we force the function to be injective, it turns out that the resulting model is the same as adding *κ* Cohen reals to the ground model, and that we have just added an enumeration of the canonical Dedekind-finite set. In the case where the function is merely surjective it turns out that we do not add any reals, sets of ordinals, or collapse any Dedekind-finite sets. This motivates the question if there is any combinatorial condition on a Dedekind-finite set *A* which characterises when a forcing will preserve its Dedekind-finiteness or not add new sets of ordinals. We answer this question in the case of ‘Adding a Cohen subset’ by presenting a varied list of conditions each equivalent to the preservation of Dedekind-finiteness. For example, 2^*A*^ is extremally disconnected, or [*A*]^<*ω*^ is Dedekind-finite.

## 1. Introduction

Cohen developed the method of forcing to prove that Cantor’s Continuum Hypothesis is not provable from the axioms of Zermelo–Fraenkel and the Axiom of Choice. He then quickly adapted the known techniques for producing models where the Axiom of Choice fails using atoms (or urelements) to match the method of forcing. His models, therefore, proved that the Axiom of Choice does not follow from the rest of the axioms of Zermelo–Fraenkel set theory. In this model, there is a canonical set of real numbers which is Dedekind-finite, that is infinite but without a countably infinite subset.

Over the years, we see time and time again how rich and interesting the theory of Cohen’s first model is. Recently, for example, Beriashvili *et al*. proved in [1] that in Cohen’s first model there is a Hamel basis for the real numbers as a vector space over the rational numbers.

From a modern perspective, Cohen’s first model is constructed by adding a countable sequence of Cohen reals, and then ‘forgetting the enumeration, while remembering the set’ using a method called symmetric extensions that extends the method of forcing and is the main tool in proving consistency results related to the Axiom of Choice. We give an overview of this technique in §2 and an overview of Cohen’s first model in §3.

In this paper, we show that forcing over Cohen’s first model can have some counterintuitive results. Our two main results to that effect are theorem 4.2, which shows that we can introduce an arbitrary enumeration of the canonical Dedekind-finite set and the resulting model is itself the appropriate Cohen extension of the ground model; and theorem 5.1 where we show that an analogue of the Levy collapse adds a surjection from the canonical Dedekind-finite set onto any fixed ordinal, but does not add new sets of ordinals. In particular, this forcing preserves Dedekind-finiteness of sets in Cohen’s first model.

Forcing (or generic) extensions of Cohen’s first model were studied by Monro in [2], where he shows that it is possible to add by a forcing extension a set which cannot be linearly ordered. This shows that, unlike the Axiom of Choice, the statement ‘every set can be a linearly ordered’ is not preserved when taking generic extensions. This line of study was extended more recently by Hall, Keremedis, and Tachtsis in [3] where the authors study Monro’s model, as well as a generic extension of their own doing, in order to show the unprovability of certain weak choice principles related to ultrafilters on *ω*.

Some of our arguments (theorem 5.1 in particular) are based on ideas in another work of Monro [4] (later developed by the first author in [5] and by Shani in [6]), where adding Cohen subsets to Dedekind-finite sets is used iteratively to prove the independence of certain weak choice principles from one another. In these works, it is crucial that no new sets of ordinals are added, and in particular no real numbers. When working over Cohen’s first model, this means that Dedekind-finiteness is preserved by adding a Cohen subset to a Dedekind-finite set.

In §6, we study Dedekind-finite sets which remain Dedekind-finite after adding a Cohen subset to them. We give 10 different equivalent conditions for this preservation, and we show that if the Dedekind-finite set is a set of real numbers, like in Cohen’s first model, then these conditions are satisfied.

## 2. Preliminaries

Since we are dealing with models of ZF and with cardinals, it is worth clarifying what we mean by cardinals. We say that a set *X can be well-ordered*, or that it is *well-orderable*, if there is an ordinal *α* and a bijection *f* : *X* → *α*. If *X* can be well-ordered, the cardinal of *X* is the smallest ordinal in bijection with *X*. If, however, *X* cannot be well-ordered, we use Scott’s trick and define its cardinal as the set $\left\{Y\in V_{\alpha }|\exists f:X\to Y\;\text{a bijection}\right\}$, where *α* is the least ordinal for which the set is not empty. The letters *κ* and *λ* will always denote well-ordered cardinals.

We say that a set *X* is *Dedekind-finite* if it has no countably infinite subset, although we will use the term Dedekind-finite exclusively to mean that it is also infinite, as finite sets already have a much shorter name. It is a simple exercise to prove that *X* is Dedekind-finite if and only if every injection *f* : *X* → *X* is a bijection. We note that if *X* is a Dedekind-finite set which can be linearly ordered, e.g. a subset of the real numbers, then [*X*]^<*ω*^ = {*a*⊆ *X*| *a*is finite} is Dedekind-finite as well. This is because every finite subset of *X* can be uniformly enumerated, so the union of countably many finite subsets will be a countable subset of *X*. Note that it is possible that *X* is a Dedekind-finite set while [*X*]^<*ω*^ is Dedekind-infinite, for example $⋃\{P_{n}|n<\omega \}$ where each *P*_*n*_ is a pair, and no infinite family of pairs admits a choice function (such a set is sometimes referred to as a Russell set, or a socks set).

Our forcing terminology is standard. We say that P is a notion of forcing if it is a partially ordered set with a maximum, $1_{P}$. We call the elements of P conditions, and we say that a condition *q* is *stronger* than a condition *p*, or that it *extends p*, if *q* ≤ *p*. We also follow Goldstern’s alphabet convention which dictates that if *p*, *q* are both conditions in P, then *p* will not denote a stronger condition than *q*. If two conditions have a joint extension we say that they are *compatible*, otherwise, they are *incompatible*. P-names are denoted by $\dot{x}$, and canonical names for ground model objects are denoted by $\overset{ˇ}{x}$ when *x* is the object in the ground model.

For a set *X*, Add(*ω*, *X*) is the forcing whose conditions are finite partial functions *p* : *X* × *ω* → 2. For a condition of *ρ*, we denote by supp(*p*) the support of *p*, which is the maximal (finite) subset of *X* such that *p* : *X* × *ω* → 2. In the context of ZF, if *A* is a set, we denote by Add(*A*, *X*) the set of partial functions *p* : *X* × *A* → 2 such that dom *p* can be well-ordered and |*p*| < |*A*|.

Finally, given a family of P-names, $\left\{{\dot{x}}_{i}|i\in I\right\}$, we denote by ${\left\{{\dot{x}}_{i}|i\in I\right\}}^{∙}$ the obvious name this family defines, that is $\left\{\langle 1_{P},{\dot{x}}_{i}\rangle |i\in I\right\}$. This notation extends to ordered pairs and to sequences as well. Using this notation, $\overset{ˇ}{x}={\left\{\overset{ˇ}{y}|y\in x\right\}}^{∙}$.

### (a) Symmetric extensions

The method of forcing, albeit very useful, preserves the Axiom of Choice when it holds in the ground model. In order to accommodate consistency proofs related to the failure of the Axiom of Choice we need to extend the method of forcing. Let P be a notion of forcing, and let *π* be an automorphism of P. Then *π* acts on P-names via this recursive definition:

$$\pi \dot{x}=\{\langle \pi p,\pi \dot{y}\rangle |\langle p,\dot{y}\rangle \in \dot{x}\}.$$

Let G be a group of automorphisms of P. We say that F is a *filter of subgroups over* G if it is a filter on the lattice of subgroups, namely it is a non-empty family of subgroups of G closed under finite intersections and supergroups. We say that F is *normal* if whenever H∈F and π∈G, $\pi H\pi^{-1}\in F$ as well.

We call ⟨P,G,F⟩ a *symmetric system* if P is a notion of forcing, G is a subgroup of Aut(P), and F is a normal filter of subgroups over G. In all cases, it is enough to require that F is a basis for a normal filter instead of a filter.

Let us fix a symmetric system for the rest of the section. We denote the group $\left\{\pi \in G|\pi \dot{x}=\dot{x}\right\}$ by ${\text{sym}}_{G}(\dot{x})$, also called the stabiliser of $\dot{x}$. We say that $\dot{x}$ is *F-symmetric* if ${\text{sym}}_{G}(\dot{x})\in F$. When $\dot{x}$ is F-symmetric, and this condition holds hereditarily for the names in $\dot{x}$, we say that $\dot{x}$ is *hereditarily* F-*symmetric*. The class ${HS}_{F}$ denotes the class of all hereditarily F-symmetric names. When the symmetric system is clear from the context, and this will always be the case, we omit the subscripts.

The forcing relation can be relativised to HS, and we use ${⊩}^{HS}$ to denote this relativization.

**Lemma (The symmetry lemma).**

*Let* p∈P *be a condition*, π∈Aut(P), *and let* $\dot{x}$ *be a* P-name, *then* $p⊩\varphi (\dot{x})\;⟺\;\pi p⊩\varphi (\pi \dot{x}).$ *Moreover, if* π∈G *and* $\dot{x}\in HS,$ *then we can replace* ⊩ *by* ${⊩}^{HS}.$

The first part of the proof appears as lemma 14.37 in [7], and the last sentence is an easy consequence of the fact that $\dot{x}\in HS$ if and only if $\pi \dot{x}\in HS$.

**Theorem.**

*Let* G⊆P *be a V*-*generic filter and let* $M={HS}^{G}=\{{\dot{x}}^{G}|\dot{x}\in HS\}.$ *Then M is a transitive class model of* ZF *in V*[*G*] *such that V*⊆ *M*.

The model *M* in the theorem is called a *symmetric extension (of *V*)*. The theorem appears in [7] as theorem 15.51.

## 3. Cohen’s first model

Cohen’s first model is the classical example of a model of set theory where the Axiom of Choice fails. This model was investigated by Halpern & Levy [8] where they prove that every set in the model can be linearly ordered, and much more. (The model is sometimes referred to as the Halpern–Levy, or the Cohen–Halpern–Levy model.) This model has many presentations in the literature (see ch. 5 in [9] for a comprehensive analysis of the construction, for example). For convenience of the reader, we give a brief overview of the construction here as well.

We assume that *V* satisfies ZFC,^1^ and we take P to be Add(*ω*, *ω*). Our group of automorphisms is given by the group of finitary permutations of *ω* acting on P in the natural way

πp(πn,m)=p(n,m),

or equivalently: *πp*(*n*, *m*) = *p*(*π*^−1^ *n*, *m*).^2^ And finally, F is the filter of subgroups generated by {fix(*E*)| *E* ∈ [*ω*]^<*ω*^}, where fix(E)={π∈G∣π↾E=id}.^3^ If $\text{fix}(E)\subseteq \text{sym}(\dot{x})$, we say that *E* is a *support* for $\dot{x}$.

For each *n*, let ${\dot{a}}_{n}$ denote the name $\left\{\langle p,\overset{ˇ}{m}\rangle |p(n,m)=1\right\}$, i.e. the canonical name for the *n*th Cohen real, and let $\dot{A}={\left\{{\dot{a}}_{n}|n<\omega \right\}}^{∙}$.

**Claim 3.1.**

If π∈G, then $\pi {\dot{a}}_{n}={\dot{a}}_{\pi n}$, therefore $\pi \dot{A}=\dot{A}$. Consequently, $\dot{A}\in HS$.

**Proposition 3.2.**

$1{⊩}^{HS}\dot{A}$ *is Dedekind-finite*.

**Proof.**

Suppose that $\dot{f}\in HS$ and $p{⊩}^{HS}\dot{f}:\overset{ˇ}{\omega }\to \dot{A}$. Let *E* be a support for $\dot{f}$, and without loss of generality supp(*p*)⊆ *E* as well. Let n∉E be some natural number, and assume towards contradiction that *q* ≤ *p* is a condition such that $q{⊩}^{HS}\dot{f}(\overset{ˇ}{m})={\dot{a}}_{n}$ for some *m* < *ω*. Let *j* < *ω* be such that j∉E∪supp(q), and let *π* be the 2-cycle (*n j*). Then the following hold:

(1) *π* ∈ fix(*E*) and therefore *πp* = *p* and $\pi \dot{f}=\dot{f}$.
(2) $\pi {\dot{a}}_{n}={\dot{a}}_{j}$.
(3) *πq* is compatible with *q*, since the only coordinates changed between *πq* and *q* are *j* and *n*, but these are mutually exclusive to the conditions.
(4) $\pi q{⊩}^{HS}\pi \dot{f}(\pi \overset{ˇ}{m})=\pi {\dot{a}}_{n}$ which is to say, by the above, $\pi q{⊩}^{HS}\dot{f}(\overset{ˇ}{m})={\dot{a}}_{j}$.

Therefore, $q\cup \pi q{⊩}^{HS}\text{`}{\dot{a}}_{n}=\dot{f}(\overset{ˇ}{m})={\dot{a}}_{j}$ and ${\dot{a}}_{n}\neq {\dot{a}}_{j}\text{'}$. This is impossible, therefore the assumption that there are such *q* and *m* must be false. Therefore, *p* forces that the range of $\dot{f}$ is finite, and in fact a subset of ${\left\{{\dot{a}}_{n}|n\in E\right\}}^{∙}$, so in particular, *p* must force that $\dot{f}$ is not injective. ▪

In the following two sections, we work in the Cohen model. We fix a *V*-generic filter G⊆P and denote by *M* the symmetric extension obtained by it and the symmetric system defined here. We will write *a*_*n*_ and *A* to denote ${\dot{a}}_{n}^{G}$ and ${\dot{A}}^{G}$, respectively.

**Remark 3.3.**

One can prove that Cohen’s model can be presented as *V*(*A*), namely the smallest transitive model of *V*[*G*] containing *V* and having *A* as an element, where G⊆P is *V*-generic; or alternatively it is ${\text{HOD}}_{V,A\cup \{A\}}^{V[G]}$, i.e. the class of all sets in *V*[*G*] which are hereditarily definable from an element of *V* and finitely many elements of *A* and *A* itself.

The idea that *V*(*A*) is the symmetric extension is relatively straightforward, and it is worth sketching the argument behind it. On the one hand, since *V*⊆ *M*, and *A* ∈ *M* we immediately have *V*(*A*)⊆ *M*. On the other hand, by analysing the proof of lemma 5.25 and lemma 5.26 in [9], we see that if *x* ∈ *M*, then we can assign to it a minimal finite subset, *A*_0_, of *A* and a name in *V* such that *A*_0_ is the copy of a support of the name, and from this we can define *x* using *A*_0_, *A* and the name from *V* as parameters. By induction on rank *x* we get that *M*⊆ *V*(*A*).

## 4. Injective collapse

For two sets *X* and *Y*, define Col_inj_(*X*, *Y*) as the partial order given by well-orderable partial injections *p* : *X* → *Y*. We note that Col_inj_(*X*, *Y*) is isomorphic to Col_inj_(*Y*, *X*). In this section, we will focus on Col_inj_(*A*, *κ*) when *κ* is an infinite ordinal,^4^ and since *A* is Dedekind-finite, the conditions are finite. It will be easier to work with Col_inj_(*κ*, *A*) instead, and as it is isomorphic to Col_inj_(*A*, *κ*), we can do that without a problem.

Let *f* : *κ* → *ω* be a finite partial injection, and let ${\dot{q}}_{f}$ denote the following name:

$${\dot{q}}_{f}={\left\{{\left\langle \overset{ˇ}{\alpha },{\dot{a}}_{f(\alpha )}\right\rangle }^{∙}|\alpha \in \text{dom}\;f\right\}}^{∙}.$$

**Claim 4.1.**

If ${\text{Col}}_{\text{inj}}{\left(\overset{ˇ}{\kappa },\dot{A}\right)}^{∙}={\left\{{\dot{q}}_{f}|f:\kappa \to \omega \text{ is a finite partial injection}\right\}}^{∙}$, then ${\left({\text{Col}}_{\text{inj}}{\left(\overset{ˇ}{\kappa },\dot{A}\right)}^{∙}\right)}^{G}={\text{Col}}_{\text{inj}}(\kappa ,A)$. In particular, if *p* forces that $\dot{q}$ is a name for a condition in Col_inj_(*κ*, *A*), then there is *p*′ ≤ *p* and *f* such that $p^{\prime }⊩\dot{q}={\dot{q}}_{f}$.

**Theorem 4.2.**

*Let F* : *κ* → *A be an M*-*generic function for Col*_inj_(*κ*, *A*). *Then F is V*-*generic for Add*(*ω*, *κ*). *In particular*, *M*[*F*] = *V*[*F*], *and all cardinals are preserved*.

Since *F* is not a filter, by *M*-generic we mean that in every dense *D*⊆Col_inj_(*κ*, *A*) in *M*, there is some *p* ∈ *D* such that *p*⊆ *F*. In other words, *F* is the function given by the generic filter.

**Proof.**

For a pair p∈P (where P=Add(ω,ω)) and a name ${\dot{q}}_{f}$, let *r*_*p*,*f*_ be the condition in Add(*ω*, *κ*) defined by *r*_*p*,*f*_(*α*, *n*) = *p*(*f*(*α*), *n*).

If $D^{*}$ is a dense subset of Add(*ω*, *κ*), then we define a name for a subset of Col_inj_(*κ*, *A*):

$$\dot{D}=\{\langle p,{\dot{q}}_{f}\rangle |\exists r\in D^{*}\text{ such that }f:\text{supp}(r)\to \omega \text{ is injective and }r=r_{p,f}\}.$$

We claim that $\dot{D}$ is a name for a dense set. Suppose that ${\dot{q}}_{f^{\prime }}$ is a name for a condition. Let *p*′ be some condition such that supp(*p*′) = rng *f*′ (we may extend *f*′ if necessary, thus strengthening ${\dot{q}}_{f^{\prime }}$), and let *r*′ = *r*_*p*′,*f*′_. By density, there is some $r\in D^{*}$ such that *r* ≤ *r*′, then we can extend *f*′ to any injective *f* and define *p* by *p*(*f*(*α*), *n*) = *r*(*α*, *n*). Then by definition, $\langle p,{\dot{q}}_{f}\rangle \in \dot{D}$ so $p⊩{\dot{q}}_{f}\in \dot{D}$. But *p* ≤ *p*′ and ${\dot{q}}_{f^{\prime }}\subseteq {\dot{q}}_{f}$. In other words, for every name ${\dot{q}}_{f^{\prime }}$ for a condition in Col_inj_(*κ*, *A*) we showed that every condition in P can be strengthened to one which forces some extension of ${\dot{q}}_{f^{\prime }}$ to be in $\dot{D}$.

Suppose now that *F* is an *M*-generic function for Col_inj_(*κ*, *A*), and let $D^{*}\in V$ be a dense open subset of Add(*ω*, *κ*). Let $\dot{D}$ be the name obtained from $D^{*}$ as above. Since *F* is *M*-generic, there is some *p* ∈ *G* and ${\dot{q}}_{f}$ such that for some $r\in D^{*}$, *r* = *r*_*p*,*f*_ and ${\dot{q}}_{f}^{G}\subseteq F$. But this means that *r*⊆ *F*, when *F* is seen as a function from *κ* × *ω* → 2, replacing each Cohen real in *A* by its characteristic function. Therefore, *F* is *V*-generic for Add(*ω*, *κ*) as wanted. Finally, since *A* = rng *F* and *M* = *V*(*A*)⊆ *V*[*F*], we have *M*[*F*] = *V*[*F*] as well. ▪

**Corollary 4.3.**

*The symmetric extension of V given by imitating Cohen’s first model, using Add*(*ω*, *κ*) (*with finitary permutations of κ*, *etc*.) *and F as in theorem 4.2 as the generic, is M*.

**Proof.**

This is true since *M* = *V*(*A*), and the argument for this equality is the same even when using Add(*ω*, *κ*), which is easy to see from analysing the same proofs as in the case *κ* = *ω*.^5^ ▪

**Remark 4.4.**

The corollary means that the process works in reverse as well, namely, starting with Add(*ω*, *λ*) with finitary permutations of *λ* and a filter of subgroups generated by pointwise stabilisers of finite subsets of *λ*, we end up with an analogue of the Cohen model where we have a set of Cohen reals which is Dedekind-finite. Using finite injective functions *f* : *κ* → *λ* provides us with the same proof as above.

This result is odd. Indeed, upon first reading, it makes no sense. It quite literally implies that there is a bijection between *ω* and *κ*. But we should point out that all it implies *inside *V** is that there is a *generic* bijection between them, i.e. we can generically collapse *κ* to be countable. Moreover, the generic objects that we always refer to are not guaranteed to exist ‘out of the blue’, rather we have a working assumption that *V* is some countable transitive model in a larger universe. And of course, under this assumption, *κ* is in fact a countable ordinal.

In the case where *κ* is singular of countable cofinality (recall that we only required that *κ* is infinite), it is well-known that adding ${ℵ}_{\omega }$ Cohen reals (to a model of CH, at least), adds ${ℵ}_{\omega +1}$ of them. When we move from *A* having order-type *ω*_1_ to *ω*_*ω*_, we seemingly add a lot more reals, which will soon disappear as we move again, say to *ω*_2_. This is the place to point out, of course, that the additional reals are the consequence of being able to define new reals from certain infinite subsequences of the generic, none of which is symmetric enough to enter the Cohen model itself.

And finally, a question.

**Question 4.5.**

It is known that the Cohen model is rather impoverished when it comes to variety of Dedekind-finite set. Indeed, every Dedekind-finite set can be taken as a subset of *ω* × [*A*]^<*ω*^. Will the results be similar if we replace *A* by any other Dedekind-finite set in the Cohen model?

One should make the obvious, and immediate, observation that taking the above question at face value the answer is no. Split *A* into two infinite parts, *A*_0_ and *A*_1_ (e.g. those reals which include 0 and those that omit it), and force with Col_inj_(*κ*, *A*_0_) instead. It is not hard to see that we do not add any enumeration of *A*_1_, which therefore remains Dedekind-finite. However, over the symmetric model that is *V*(*A*_0_), the result was indeed the same as above.

## 5. ‘Levy collapse’ without adding reals (or sets of ordinals)

For two sets *X* and *Y*, denote by Col(*X*, *Y*) the set of partial functions *p* : *X* → *Y* such that |*p*| is well-ordered and |*p*| < |*X*|, ordered by reverse inclusion. This coincides with the standard definition when *X* can be well-ordered, but we will focus on the case where *X* = *A* in Cohen’s first model, meaning that the conditions are, as before, finite functions.

In a manner similar to claim 4.1, if *q* ∈ Col(*A*, *κ*) is a condition, where *κ* is some well-ordered cardinal, then there is some *f* : *ω* → *κ* in *V* such that $q={\dot{q}}_{f}^{G}$, with ${\dot{q}}_{f}$ defined as in §4.

**Theorem 5.1.**

*Let κ be an infinite cardinal and let G*⊆*Col*(*A*, *κ*) *be an M*-*generic filter. Then M and M*[*G*] *have the same sets of ordinals*.

**Proof.**

Let $\dot{X}\in M$ be a Col(*A*, *κ*)-name for a set of ordinals. It is easier to consider $\dot{X}$ as a name in the iteration $P*\text{Col}{\left(\dot{A},\overset{ˇ}{\kappa }\right)}^{∙}$, whose projection to a P-name of a Col(*A*, *κ*)-name, denoted by $\left[\dot{X}\right]$, is in HS.^6^ Moreover, since we have such canonical names for conditions in Col(*A*, *κ*), and we are only interested in this iteration of two steps, we may assume that the conditions in this iteration have the form $\left\langle p,{\dot{q}}_{f}\right\rangle$.

Note that if π∈G, then *π* acts on $P*\text{Col}{\left(\dot{A},\overset{ˇ}{\kappa }\right)}^{∙}$ in the obvious way

$$\pi \langle p,{\dot{q}}_{f}\rangle =\langle \pi p,\pi {\dot{q}}_{f}\rangle =\langle \pi p,{\dot{q}}_{f\circ \pi }\rangle .$$

Let $\left\langle p,{\dot{q}}_{f}\right\rangle$ be a condition which forces that $\dot{X}$ is a name for a set of ordinals, and let *E* be a support for $\dot{X}$, i.e. a finite subset of *ω* for which $\text{fix}(E)\subseteq \text{sym}([\dot{X}])$. We may assume that supp(*p*) = *E* = dom *f*. Suppose that $\left\langle p_{0},{\dot{q}}_{f_{0}}\right\rangle$ and $\left\langle p_{1},{\dot{q}}_{f_{1}}\right\rangle$ are two extensions of $\left\langle p,{\dot{q}}_{f}\right\rangle$. Again, we may assume that supp(*p*_*i*_) = dom *f*_*i*_ for *i* < 2.

If $p_{1}↾E=p_{2}↾E$, then the two must agree on any statement of the form $\overset{ˇ}{\alpha }\in \dot{X}$. This is because there is an automorphism in fix(*E*) moving $\text{supp}(p_{0})\setminus E$ to be disjoint of supp(*p*_1_), which means that $\left\langle \pi p_{0},\pi {\dot{q}}_{f_{0}}\right\rangle$ is compatible with $\left\langle p_{1},{\dot{q}}_{f_{1}}\right\rangle$ while $\pi \overset{ˇ}{\alpha }=\overset{ˇ}{\alpha }$ and $\pi \dot{X}=\dot{X}$. Here, we used the fact that dom *f* = *E*, as well as the fact the conditions are not injective. Indeed, for injective functions agreeing on their common domain is not enough to be compatible.

In particular, if $\left\langle p^{\prime },{\dot{q}}_{f^{\prime }}\rangle \leq \langle p,{\dot{q}}_{f}\right\rangle$ and $\langle p^{\prime },{\dot{q}}_{f^{\prime }}\rangle ⊩\overset{ˇ}{\alpha }\in \dot{X}$, then $\left\langle p^{\prime }↾E,{\dot{q}}_{f^{\prime }↾E}\rangle =\langle p^{\prime }↾E,{\dot{q}}_{f}\right\rangle$ already forced this statement, and the same for $\overset{ˇ}{\alpha }\notin \dot{X}$, of course. Therefore, the conclusion follows, and therefore $\dot{X}$ is a name for a set in *M*, given by the name ${\dot{X}}_{f}=\{\langle p^{\prime },\overset{ˇ}{\alpha }\rangle |\langle p^{\prime }↾E,{\dot{q}}_{f}\rangle ⊩\overset{ˇ}{\alpha }\in \dot{X}\}$. ▪

This provides another proof of the known fact (see Problem 16 in ch. 5 of [9]) that two models of ZF with the same sets of ordinals are not necessarily equal.^7^

**Corollary 5.2.**

*Forcing with Col*(*A*, *κ*) *over M preserves cofinalities*.

**Corollary 5.3.**

*A is still Dedekind-finite after forcing with Col*(*A*, *κ*).

**Proof.**

Suppose not, then there is an injective function *f* : *ω* → *A* in *M*[*H*], where *H* is *M*-generic for Col(*A*, *κ*), and *f* can be coded as a real, namely a subset of *ω*. However, by theorem 5.1 no new reals are added, and therefore *f* ∈ *M*. This is a contradiction since *A* is Dedekind-finite in *M*. ▪

**Corollary 5.4.**

*Every Dedekind-finite set remains Dedekind-finite after forcing with Col*(*A*, *κ*).

**Proof.**

There is an injection from every set in *M* into [*A*]^<*ω*^ × *η* for some ordinal *η*, therefore for a Dedekind-finite set in *M* we can take *η* = *ω*. But this means that if *A* remains Dedekind-finite, so must [*A*]^<*ω*^, as *A* is a set of real numbers, and therefore every other Dedekind-finite set remains Dedekind-finite as well. ▪

In *M*, the set *A* has a partition into ${ℵ}_{0}$ different parts (e.g. by looking at min *a* for *a* ∈ *A*, which by genericity must obtain each possible value infinitely many times). After forcing with Col(*A*, *κ*) we added new partitions of size *κ*, without adding new sets of ordinals. This is in contrast to the results of Monro in [2]: in his model, the generic partition is an infinite partition of *A* which itself cannot be split into two infinite sets making the partition Dedekind-finite.

**Question 5.5.**

In the previous section and in this one, the proofs involved in a fairly meaningful way the Cohen forcing itself. What happens when we consider a similar symmetric extension obtained by using a different kind of real numbers, e.g. random reals, Sacks reals, etc., or even a mixture of these? On its face it seems that the proof uses more of the fact that we take a finite-support product of infinitely many copies of the same forcing, rather than the specific properties of the Cohen forcing. To what extent can this be pushed?

## 6. Adding a Cohen subset to a Dedekind-finite set

Corollary 5.3 shows that the Dedekind-finite set *A* in Cohen’s first model is still Dedekind-finite after forcing with Col(*A*, *κ*), and this leads to the problem of finding more general condition that ensure this. In this section, we provide characterisations, in ZF, of those Dedekind-finite sets *A* that remain Dedekind-finite after forcing with Add(*A*, 1) = Col(*A*, 2). The combined results can already be stated in the following result, although some of the notions in the theorem are only defined below.

**Theorem 6.1.**

*Let A be a Dedekind-finite set. The following are equivalent*:

(*1*) [*A*]^<*ω*^ *is Dedekind-finite*.
(*2*) *Add*(*A*, 1) *contains no infinite antichains.*
(*3*) *Add*(*A*, 1) *contains no countably infinite antichains*.
(*4*) *Add*(*A*, 1) *has the finite decision property*.
(*5*) *A remains Dedekind-finite in any generic extension by Add*(*A*, 1).
(*6*) *A is not collapsed in any generic extension by Add*(*A*, 1).
(*7*) *Add*(*A*, 1) *fails to add a Cohen real*.
(*8*) *Add*(*A*, 1) *fails to add a real*.
(*9*) *Add*(*A*, 1) *fails to add a set of ordinals*.
(*10*) 2^*A*^ *is extremally disconnected*.

Some of these conditions (e.g. (1)) already imply that *A* is Dedekind-finite, but others do not (e.g. (7) holds for *A* = *ω*_1_ when assuming ZFC, and (6) holds for *A* = *ω* even in ZF).

The forcing Add(*A*, 1) was recently studied by Goldstern & Klausner [10] where the authors study the possible effects of this forcing on the structure of cardinals, as well as some specific properties of the forcing, e.g. condition (2) in our theorem, that occur when *A* is assumed to be Dedekind-finite with particular properties.

Our theorem admits an easy corollary, which is applicable to Cohen’s first model.

**Corollary 6.2.**

*If A is a Dedekind-finite set which can be linearly ordered, in particular a set of real numbers, then all the conditions of theorem 6.1 hold*.

In the proofs, we will use the *sunflower lemma*, a finite version of the Δ-system lemma. Before we state the lemma, we fix the following notation. A *sunflower* is a collection of sets, *S*, such that for some *t*, u∩v=t for all *u* ≠ *v* in *S*. Moreover, the set *t* is called the *centre* of the sunflower.

**Lemma 6.3 (Erdős–Rado [11]).**

*If a and b are positive integers, then any collection of b*!*ab* + 1 *sets of size* ≤*b contains a sunflower of size* >*a*.

The above lemma, involving only finite sets, is of course provable without the Axiom of Choice. Less obvious, though, is that the following lemma can also be proved in ZF, as was done by Keremedis *et al*. [12].^8^

**Lemma 6.4 (Lemma 4 in [12]).**

*If X is an infinite collection of sets of size b*, *for a natural number b* > 1, *then there is a finite* t⊆⋃X *such that for every positive number a*, *there is a subset of X of size a which is a sunflower with centre t*.

It is easy to see that [*A*]^<*ω*^ is Dedekind-infinite if and only if there is a sequence ***A*** = 〈*A*_*n*_| *n* < *ω*〉 of disjoint non-empty finite subsets of *A*. We will call such a sequence a *disjoint sequence*. We fix some more notation: for any *K*⊆Add(*A*, 1) we define supp^*K*^ = {dom *p*| *p* ∈ *K*}. Note that *K* is finite if and only if supp^*K*^ is finite.

**Lemma 6.5.**

*The following are equivalent*:

(*a*) *Add*(*A*, 1) *contains a countably infinite antichain*.
(*b*) *Add*(*A*, 1) *contains an infinite antichain*.
(*c*) [*A*]^<*ω*^ *is Dedekind-infinite*.

**Proof.**

(a)  ⟹  (b) is clear.

(b)  ⟹  (c): Suppose that Add(*A*, 1) contains an infinite antichain *C*. We show that for all *k* < *ω*, *C*_*k*_ = {*p* ∈ *C*| |dom *p*| = *k*} is finite. It then follows that $D=\underset{k<\omega }{⋃}{\text{supp}}^{C_{k}}$ is an infinite union of finite sets and hence [*A*]^<*ω*^ is Dedekind-infinite. Towards a contradiction, suppose that *C*_*k*_ is infinite for some *k* < *ω*. Then ${\text{supp}}^{C_{k}}$ contains arbitrarily large sunflowers by lemma 6.3. Since their centres are all of size at most *k*, we can find two compatible conditions in *C*_*k*_, contradicting the assumption that *C* is an antichain.

(c)  ⟹  (a): If [*A*]^<*ω*^ is Dedekind-infinite, fix a disjoint sequence 〈*A*_*n*_| *n* < *ω*〉 and $B_{n}=\underset{i\leq n}{⋃}A_{i}$. Define *p*_0_ : *B*_0_ → {0, 1} to take the value 1 on *B*_0_, and for n∈ω∖{0}, define *p*_*n*_ : *B*_*n*_ → {0, 1} to take value 1 on *A*_*n*_ and 0 on *B*_*n*−1_. Then *C* = {*p*_*n*_| *n* < *ω*} is a countably infinite antichain. ▪

The equivalence of (a) and (c) was independently proved recently by Keremedis and Tachtsis, see lemma 1 in [13]. They prove in theorem 3 a further equivalence in terms of the topological *cellularity* of the space 2^*A*^.

**Definition 6.6.**

We say that Add(*A*, 1) has the *finite decision property* if for all formulae $\varphi (\dot{x})$, the set $M^{\varphi (\dot{x})}$ of minimal elements of $N^{\varphi (\dot{x})}=\{p|p⊩\varphi (\dot{x})\}$ with respect to restriction is finite.

**Lemma 6.7.**

*The following are equivalent*.^9^

(*a*) *Add*(*A*, 1) *has the finite decision property*.
(*b*) [*A*]^<*ω*^ *is Dedekind-finite*.

**Proof.**

(a)  ⟹  (b): Suppose that [*A*]^<*ω*^ is Dedekind-infinite, and let 〈*A*_*n*_| *n* < *ω*〉 be a disjoint sequence witnessing that. Let $\dot{x}$ be the Add(*A*, 1)-name for the least *n* such that the canonical generic subset of *A* contains *A*_*n*_. Let $\varphi (\dot{x})$ denote a formula stating that $\dot{x}$ is even. It is then easy to see that $M^{\varphi (\dot{x})}$ is infinite.

(b)  ⟹  (a): Suppose that $\varphi (\dot{x})$ is a formula such that $M^{\varphi (\dot{x})}$ is infinite. We can assume that $M_{k}^{\varphi (\dot{x})}=\{p\in M^{\varphi (\dot{x})}||\text{dom}\;p|=k\}$ is infinite for some *k* < *ω*, since one can otherwise construct a disjoint sequence. Let $M=M_{k}^{\varphi (\dot{x})}$. Since *M* is infinite, supp^*M*^ is infinite as well. By lemma 6.4, there is some finite $t\subseteq ⋃{\text{supp}}^{M}$ which is the centre of arbitrarily large sunflowers in supp^*M*^, fix such *t*.

There are only finitely many possible values that a condition can take on *t*, in fact at most 3^*k*^, where the third value stands for ‘undefined’. Therefore, there is a condition *q* with the property: there are arbitrarily large subsets *K* of *M* such that supp^*K*^ forms a sunflower with centre *t* and p↾t=q for all *p* ∈ *K*. Since *q* is a proper subset of *p* if |*K*| > 1, it must be that $q\text{⧸}⊩\varphi (\dot{x})$, by the minimality of *p*.

**Subclaim.**

$q⊩\varphi (\dot{x})$.

**Proof.**

Otherwise take some *r* ≤ *q* with $r⊩\neg \varphi (\dot{x})$. Then *r* is incompatible with all elements of *M*. Moreover, take a subset *K* of *M* as above with |*K*| > |*r*|. Since supp^*K*^ forms a sunflower with centre *t* and |*K*| > |*r*|, there is some *s* ∈ *K* with dom r∩dom s=t. We further have s↾t=q by the choice of *K*. Therefore, *r* and *s* are compatible. But this contradicts the fact that *r* and *s* force opposite truth values of $\varphi (\dot{x})$. □

But this is a contradiction, as $q\text{⧸}⊩\varphi (\dot{x})$. Therefore, $M_{k}^{\varphi (\dot{x})}$ is finite for all *k*, and we can construct a disjoint subset as wanted. ▪

**Definition 6.8.**

A set *A* is *collapsed* in an outer model *W* of *V* if there is a subset of *A*, *B* ∈ *V*, such that V⊨|B|<|A|, but W⊨|B|=|A|.

**Lemma 6.9.**

*Let A be a Dedekind-finite set. The following are equivalent*.

(*a*) [*A*]^<*ω*^ *is Dedekind-infinite*.
(*b*) *A is Dedekind-infinite in any generic extension by Add*(*A*, 1).
(*c*) *A is collapsed in any generic extension by Add*(*A*, 1).

**Proof.**

(a)  ⟹  (b): Assume that [*A*]^<*ω*^ is Dedekind-infinite, and let 〈*A*_*n*_| *n* < *ω*〉 be a disjoint sequence witnessing that. Let *G*⊆Add(*A*, 1) be a *V*-generic filter, and let *B* be {a∣∃p∈G,p(a)=1}. Define *f*(*n*) = *a* whenever $B\cap A_{n}=\{a\}$, which by a density argument happens infinitely often. This defines an injection from an infinite subset of *ω* into *A*, and therefore *A* is Dedekind-infinite.

(b)  ⟹  (c): Suppose that *A* is Dedekind-infinite in the generic extension, and let *f* : *A* → *A* be an injection which is not a bijection, then there is some *a* ∈ *A* such that *f*(*x*) ≠ *a* for all *x* ∈ *A*. In *V*, by Dedekind-finiteness of *A* there, the set A∖{a} is strictly smaller in size, and thus witnessing that *A* was collapsed.

(c)  ⟹  (b): Trivial.

(b)  ⟹  (a): Assume towards a contradiction that [*A*]^<*ω*^ is Dedekind-finite, but suppose that $p⊩\dot{f}:\overset{ˇ}{\omega }\to \overset{ˇ}{A}$ is injective, and for simplicity, assume that p=1. Let $\varphi (\overset{ˇ}{n},\overset{ˇ}{a})$ denote the formula $\dot{f}(\overset{ˇ}{n})=\overset{ˇ}{a}$. Let $s_{a}^{n}$ be the set ${\text{supp}}^{M^{\varphi (\overset{ˇ}{n},\overset{ˇ}{a})}}$ for *n* < *ω* and *a* ∈ *A*. By the finite decision property in lemma 6.7, $M^{\varphi (\overset{ˇ}{n},\overset{ˇ}{a})}$ and thus $s_{a}^{n}$ are finite.

**Subclaim.**

For any *n* < *ω*, $\left\{a\in A|s_{a}^{n}\neq \emptyset \right\}$ is finite.

**Proof.**

Fix *n* < *ω* and let *B* denote $\left\{a\in A|s_{a}^{n}\neq \emptyset \right\}$. Towards a contradiction, suppose that *B* is infinite. First assume that for all *k* < *ω*, there are only finitely *a* ∈ *B* with $|s_{a}^{n}|=k$. Then *A*_*k*_ defined as $\underset{|s_{a}^{n}|=k}{⋃}s_{a}^{n}$ is finite as well. Since the sets $M^{\varphi (\overset{ˇ}{n},\overset{ˇ}{a})}$ are disjoint as *a* ∈ *A* varies, 〈*A*_*k*_| *k* < *ω*〉 has an injective infinite subsequence and hence [*A*]^<*ω*^ is Dedekind-infinite.

Now assume that for some *k* < *ω*, there are infinitely many *a* ∈ *B* with $|s_{a}^{n}|=k$. Note that *k* ≠ 0 by the definition of *B*. Since the sets $M^{\varphi (\overset{ˇ}{n},\overset{ˇ}{a})}$ are disjoint as *a* ∈ *A* varies, the set $S=\{s_{a}^{n}|a\in B\}$ is infinite. This set contains arbitrarily large sunflowers by lemma 6.3.

Let *T* be a sunflower in *S* of size 3^*k*^ + 1. Since the centre of *T* has size ≤*k*, there are at most 3^*k*^ possible values for restrictions to the centre of *T*. Hence we can find *a* ≠ *b* in *B* and conditions $p\in M^{\varphi (\overset{ˇ}{n},\overset{ˇ}{a})}$ and $q\in M^{\varphi (\overset{ˇ}{n},\overset{ˇ}{a})}$ with *p* compatible with *q*. But this contradicts the fact that conditions in $M^{\varphi (\overset{ˇ}{n},\overset{ˇ}{a})}$ and $M^{\varphi (\overset{ˇ}{n},\overset{ˇ}{b})}$ are pairwise incompatible. ▪

Note that $s_{a}^{n}=\emptyset$ implies that $M^{\varphi (\overset{ˇ}{n},\overset{ˇ}{a})}$ is either empty or contains only 1. Thus the subclaim implies that *M*^*n*^ defined as $\underset{a\in A}{⋃}M^{\varphi (\overset{ˇ}{n},\overset{ˇ}{a})}$ is finite for all *n* < *ω*. In other words, there are only finitely many possible choices for $\dot{f}(n)$. However, 1 forces that $\dot{f}$ has infinite range and thus $\underset{n<\omega }{⋃}M^{n}$ is an infinite subset of *A*. This allows us to construct a disjoint sequence in [*A*]^<*ω*^, in contradiction to the assumption that [*A*]^<*ω*^ is Dedekind-finite. ▪

Note that by the homogeneity of Add(*A*, 1) the above proof that (b) implies (a) does not depend on the choice of a generic filter, and since collapsing *A* or making it Dedekind-infinite can be stated as a formula whose free variables are all canonical ground model names, there is no dependence on any specific condition either.

**Lemma 6.10.**

*Let A be a Dedekind-finite set. The following are equivalent*.

(*a*) [*A*]^<*ω*^ *is Dedekind-infinite*.
(*b*) *Add*(*A*, 1) *adds a Cohen real*.
(*c*) *Add*(*A*, 1) *adds a real*.
(*d*) *Add*(*A*, 1) *adds a set of ordinals*.

**Proof.**

(a)  ⟹  (b): Let 〈*A*_*n*_| *n* < *ω*〉 be a disjoint sequence witnessing that [*A*]^<*ω*^ is Dedekind-infinite. Let $\dot{x}=\{\langle p,\overset{ˇ}{n}\rangle |p[A_{n}]=\{0\}\}$. A density argument shows that $\dot{x}$ is a name for a Cohen real.

(b)  ⟹  (c)  ⟹  (d) is trivial.

(d)  ⟹  (a): Suppose that 1 forces that $\dot{X}$ is a new subset of some ordinal *η*, and let $\varphi (\overset{ˇ}{\alpha })$ denote the formula $\overset{ˇ}{\alpha }\in \dot{X}$. By the finite decision property in lemma 6.7, $M^{\varphi (\overset{ˇ}{\alpha })}$ is finite for all *α* < *η*. However, the union of the domains of conditions in $\underset{\alpha <\eta }{⋃}M^{\varphi (\overset{ˇ}{\alpha })}$ is infinite, since $\dot{X}$ is a name for a new set of ordinals. Thus it is easy to construct a disjoint sequence in [*A*]^<*ω*^. ▪

We equip 2^*A*^ with the product topology. Moreover, let *N*_*p*_ = {*x* ∈ 2^*A*^| *p*⊆ *x*} denote the basic open set associated with *p* ∈ Add(*A*, 1). Note that 2^*A*^ is a Hausdorff space. We will consider the following notion: a topological space is called *extremally disconnected* if the closure of every open set is open. Note that for Hausdorff spaces, this strengthens the property of being *totally disconnected*.

**Lemma 6.11.**

*The following are equivalent*.

(*a*) 2^*A*^ *is extremally disconnected*.
(*b*) [*A*]^<*ω*^ *is Dedekind-finite*.

**Proof.**

(a)  ⟹  (b): Suppose that [*A*]^<*ω*^ is Dedekind-infinite. We will show that 2^*A*^ is not extremally disconnected. To see this, let 〈*A*_*n*_| *n* < *ω*〉 be a disjoint sequence and $B_{n}=\underset{i\leq n}{⋃}A_{i}$. Define *p*_0_ : *B*_0_ → {0, 1} to take the value 1 on *B*_0_, and for n∈ω∖{0}, define *p*_*n*_ : *B*_*n*_ → {0, 1} to take value 1 on *A*_*n*_ and 0 on *B*_*n*−1_. Then *U* defined as the union $\underset{n<\omega }{⋃}N_{p_{n}}$ is open. But the closure of *U* is U∪{0} which is not open, since it does not contain any open neighbourhood of **0**, where **0** denotes the constant function **0**(*a*) = 0 for all *a* ∈ *A*.

(b)  ⟹  (a):^10^ Suppose that [*A*]^<*ω*^ is Dedekind-finite. Let *N*_*I*_ be an open set of 2^*A*^ given by the union $\underset{p\in I}{⋃}N_{p}$. We will show that its closure is open.

Note that *f* ∈ 2^*A*^ fails to be in the closure of *N*_*I*_ if and only if there is some *p* ∈ Add(*A*, 1) such that *p*⊆ *f* and $N_{p}\cap N_{I}=\emptyset$, which equivalently means that $p⊩\dot{g}\notin {\dot{N}}_{I}$, where $\dot{g}$ is the canonical generic function *A* → {0, 1} and ${\dot{N}}_{I}$ is the re-interpretation of the union $\underset{p\in I}{⋃}N_{p}$ in the generic extension. But by the finite decision property, which holds by lemma 6.7, there is a finite set, *M*, of minimal elements *p* such that $p⊩\dot{g}\notin {\dot{N}}_{I}$. In particular, $C=\underset{p\in M}{⋃}N_{p}$ is a finite union of clopen sets and thus closed. So the closure of *N*_*I*_ is open, as wanted. ▪

We finish with a question of interest, as one should.

**Question 6.12.**

As we observed at the beginning of this section, Add(*A*, 1) is the same as Col(*A*, 2). Moreover, we proved in the previous section that Col(*A*, *κ*) does not make *A* Dedekind-infinite, where *A* is the canonical Dedekind-finite set in Cohen’s first model. Is there a similar characterisation as in theorem 6.1 for Col(*A*, *κ*), or even more generally for an arbitrary forcing that adds a fresh subset to a Dedekind-finite set?

## Footnotes

1 Traditionally, *V* is taken as *L*, but this is not important.

2 While the latter definition seems more natural from a naive point of view, it is in fact the former that is easier to work with.

3 Because we work with pointwise stabilisers of finite sets, using the full symmetry group of *ω* is the same as using finitary permutations.

4 We can of course assume it is a cardinal, but the assumption is never used.

5 We can also obtain the same by applying Feferman’s theorem appearing as Problem 23 in ch. 5 of [9] instead of the proof analysis.

6 While this is quite simple to understand in this case, a more general theory of iterations of symmetric extensions was developed by the first author in [5].

7 This requires, of course, that the Axiom of Choice fails in both models.

8 We can actually use this lemma instead of the Erdős–Rado lemma in the arguments in this paper.

9 One can easily formulate this lemma and its proof without using forcing. Then $N^{\varphi (\dot{x})}$ is replaced by any subset *N* of Add(*A*, 1) with the following property: if *M* is a subset of *N* that is dense below some *p* ∈ *N*, then *p* ∈ *M*.

10 This argument, like lemma 6.7, can be made without forcing. Here, we can use the topology.

## Data accessibility

All data are available in the paper and references.
